# Intraoperative Hypotension Prediction Model Based on Systematic Feature Engineering and Machine Learning

**DOI:** 10.3390/s22093108

**Published:** 2022-04-19

**Authors:** Subin Lee, Misoon Lee, Sang-Hyun Kim, Jiyoung Woo

**Affiliations:** 1 Bigdata Engineering Department, SCH Media Labs, Soonchunhyang University, Asan 31538, Korea; lsb102030@naver.com; 2 Department of Anesthesiology and Pain Medicine, Soonchunhyang University Bucheon Hospital, Soonchunhyang University College of Medicine, Bucheon 14584, Korea; misoonlee@schmc.ac.kr (M.L.); skim@schmc.ac.kr (S.-H.K.)

**Keywords:** machine learning, vital sign, invasive blood pressure, feature engineering, hypotension, arterial hypotension

## Abstract

Arterial hypotension is associated with incidence of postoperative complications, such as myocardial infarction or acute kidney injury. Little research has been conducted for the real-time prediction of hypotension, even though many studies have been performed to investigate the factors which affect hypotension events. This forecasting problem is quite challenging compared to diagnosis that detects high-risk patients at current. The forecasting problem that specifies when events occur is more challenging than the forecasting problem that does not specify the event time. In this work, we challenge the forecasting problem in 5 min advance. For that, we aim to build a systematic feature engineering method that is applicable regardless of vital sign species, as well as a machine learning model based on these features for real-time predictions 5 min before hypotension. The proposed feature extraction model includes statistical analysis, peak analysis, change analysis, and frequency analysis. After applying feature engineering on invasive blood pressure (IBP), we build a random forest model to differentiate a hypotension event from other normal samples. Our model yields an accuracy of 0.974, a precision of 0.904, and a recall of 0.511 for predicting hypotensive events.

## 1. Introduction

Arterial hypotension that occurs during anesthesia may increase the incidence of postoperative complications, such as myocardial infarction or acute kidney injury [1]. Careful monitoring of the patient’s hemodynamic changes is required during anesthesia, and when hypotension is detected, immediate treatment is provided to maintain hemodynamic stability. If the patient’s hemodynamic changes are predicted in advance, it will be possible to provide safer anesthesia to the patient by maintaining hemodynamic stability. Most patient monitor devices that monitor a patient’s vital signs store the data for a short time [2], and the data are mostly deleted without being utilized for other purposes.

However, these vital sign data can be useful in developing a tool which can predict a patient’s hemodynamic changes.

While research on hypotension in operation room mostly focuses on investigating the factors affecting a hypotension event, not much research has been performed on real-time prediction of hypotension. The advanced warning that hypotension is imminent at least 5 min ahead enables clinicians to take proper measures to reduce the impact of hypotension. This forecasting problem is quite challenging compared to diagnosis that detects high-risk patients at current. The forecasting problem that does not specify when the event occurs is easier than the forecasting problem that specifies the event time. Furthermore, it is very difficult to advance the predictable time compared to the event occurrence time. In this work, we will challenge the forecasting problem in 5 min advance. 

Previous works on hypotension prediction have proposed various indices that originate from the waveform of vital signs. Recently, machine learning algorithms have replaced the scoring system, identified significant factors, and measured their effect on an event automatically.

In this study, we propose a systematic feature engineering that is applicable to any kind of vital signs and build a machine learning model that predicts hypotension in advance. We aim to build a simple model that does not require many vital signs and only requires invasive blood pressure (IBP). Instead of hand-crafted features on IBP, we propose a common feature extraction model that can be applicable to various kinds of vital signs. The feature extraction model includes the statistical analysis, peak analysis, change analysis, and frequency analysis. We build an ensemble model using a random forest model to handle numerous features in heterogenous samples.

## 2. Related Works

Many studies using vital signs have been performed in the intensive care unit (ICU); however, there is little research for the operation room where vitality is relatively constant compared to ICU [3,4,5,6,7,8,9].

Recently, studies that predict hypotension, depth of anesthesia, hypothermia, etc., have been conducted in the operating room. Topics of the studies using vital signals during surgery encompass estimation of the depth of anesthesia, estimation of blood pressure, event prediction regarding blood pressure, and heart failure. The former models were designed to predict whether a patient would suffer an event or not at the initial stage of operation [10,11,12,13,14]. These works can inform high-risk patients, but are limited in alerting an alarm for real-time treatment for an event. The recent prediction models are developed into real-time prediction models and the number of works is limited. We briefly reviewed real-time models in terms of classification and regression.

### 2.1. Real-Time Event Detection

Yang et al. [15] reported a convolutional neural network (CNN)-based deep learning model that predicts the stroke volume with a 20 s arterial blood pressure waveform. Lee et al. [16] created a CNN-based deep learning model to predict hypotension before 5 min, 10 min, and 15 min, respectively, using IBP, electrocardiography (ECG), photoplethysmography (PPG), and capnography (CO_2_). They demonstrated that the precision and recall were higher than our research, but their experimental setting was different from ours. They included only the period where non-hypotension lasted for 20 min only. Their environment was less realistic because their model did not work on samples that included any data below the criteria. In addition, it is not sure that they focused on predictions for the very first time point of hypotension. As hypotension occurred, an alarm given in a timely manner was required in the first place. Chen and Qi [17] proposed a feature-based model. They predicted heart failure by statistical features; textualization; and imaging using HR, SBP, DBP, SpO_2_, and pulse pressure difference (PP). Among the statistical feature models, the gradient boosting tree model had the highest accuracy of 84%, while textualization and imaging models had accuracies of 81% and 83% for the logistic regression and convolution neural network models, respectively. Furthermore, in predicting heart failure, the statistical feature-based model gave the best results. The statistical features used in this study included the mean; variance; minimum; maximum; 25%, 50%, and 75% quantiles; skewness; kurtosis; and first-order difference of each feature.

These real-time detection models suffer from the class imbalance problem and rarely achieve good performance. Most works set up an artificial environment to make the models work. 

### 2.2. Real-Time Regression

The following works have been proposed to real-time regression for blood pressure or depth of anesthesia. The real-time regression model showed better performance compared to the event detection model because regression models are free from the class imbalance problem that the event detection model suffers from. This imbalance problem makes the model difficult to generalize. The models adopted in previous works were developed from machine learning models incorporated with feature engineering to the deep learning model. RNN-based models suitable for time sequence were adopted, and CNN models suitable for imaging were also adopted after the vital sign transformed into an image. 

Regarding the model adopted machine learning with feature engineering, Jeong et al. [18] developed a blood pressure prediction model by applying the deep learning model to non-invasive blood pressure and other vital signs. This work proposed a concise model using derived variables rather than the original waveform data.

Gopalswamy et al. [10] proposed a long short-term memory (LSTM) model to predict intraoperative blood pressure and length of stay (LOS) using temperature, respiratory rate (RR), heart rate (HR), diastolic blood pressure (DBP), systolic blood pressure (SBP), fraction of inspired O_2_ (FiO_2_), and end-tidal CO_2n_ (EtCO_2_). Sadrawin et al. [1] reported artificial neural networks (ANNs) which can predict the depth of anesthesia using electroencephalography (EEG), electromyography (EMG), HR, pulse, SBP, DBP, and signal quality index (SQI). Regarding CNN models, Liu et al. [19] presented a CNN model that can predict the depth of anesthesia by transforming the EEG signal into a spectral image through modified short-time Fourier transform (STFT) transformation. Chowdhury et al. [20] demonstrated that a deep learning model can predict the depth of anesthesia by imaging the ECG and PPG signals as a heat map.

### 2.3. Research Gaps

From the literature review, we found several research gaps:

- Little research has been conducted using the vital signs collected in the operation room, while plenty of research has been carried out in ICU.
- Previous works focusing on the vital signs in the operation room deal with the depth of anesthesia. Rare events such as hypotension are important for patient health.
- Most studies focus on diagnoses that can identify high-risk patients who will suffer an event rather than prognosis. To react to the event in a preventive way, a real-time prediction model is required.
- Light-weight real-time prediction models are more effective for instant answering. However, existing works used many kinds of vital sign [14,15,18,19,21].

## 3. Materials and Methods

### 3.1. Patient Population

The data used in this paper were collected in Soonchunhyang University Bucheon Hospital through the Vital Recorder [21] program, which used the Bx50 monitor for patients whose blood pressure was measured with intra-arterial catheters (ART) during operations. These data were based on the continuous monitoring of blood pressure as IBP and were collected from 30 December 2019 to 30 October 2020 using an IBP time series of 888 patients. IBP data were recorded in units of 100 Hz.

### 3.2. Preprocessing

A moving average was widely used to smooth data and remove short-term fluctuations to highlight the patterns embedded in time sequences. High-resolution data naturally exhibit fluctuations, making patterns distorted and feature extraction difficult.

To derive samples from waveform IBP, we set the specific feature observation period, delay period, and event observation period, accordingly. The feature observation period refers to the period where features are extracted, the delay period refers to how far into the future the forecasting targets, and the event observation period is when the event is observed.

For our model, the observation period was set as 20 s, the delay period was set as 5 min to provide enough time for medical staff to react, and the event observation period was set as 1 min. To differentiate the samples related to hypotension from normal samples, the observation period was kept as short as possible. However, the frequency-based features required many time points. Thus, we compromised these two contradictions and set up the observation period as 20 s. In other works, the observation period was set to 30 s. We aimed to vary the observation period up to 30 s and check the performance. The class information was retrieved over a 1 min observation period. A class observation period was set up instead of picking a point, though this was not due to difficulties in characterizing a certain point. The class observation period was long enough to generate more samples for the hypotension event. In our future work, we aim to perform various experiments with varying observation and class observation periods. 

A hypotension sample is defined as a case where the maximum value of a 2 s moving average of IBP during the class observation period falls short of 65 mmHg. A normal sample is defined as a case where the minimum value of a 2 s moving average of IBP during 1 min exceeds 65 mmHg. Blood pressure data were used for feature extraction during the observation period. We excluded samples associated with hypotensive events which occur during the observation window or the delay period; otherwise, it would be unnecessary to make the prediction. 

Any sample that satisfied the hypotension event during the data observation and delay periods were also excluded. In addition, if the hypotensive event occurred consecutively, only the first event needed to be considered. This specifically relates to cases with a maximum value of the 2 s moving average of the data combined with the observation section, whereby the delay section is <65 was excluded. This aimed to make a prediction at least 5 min in advance, except for cases where hypotension was predicted in a situation with hypotension. The results of preprocessing are shown in Table 1.

**Figure 1:**
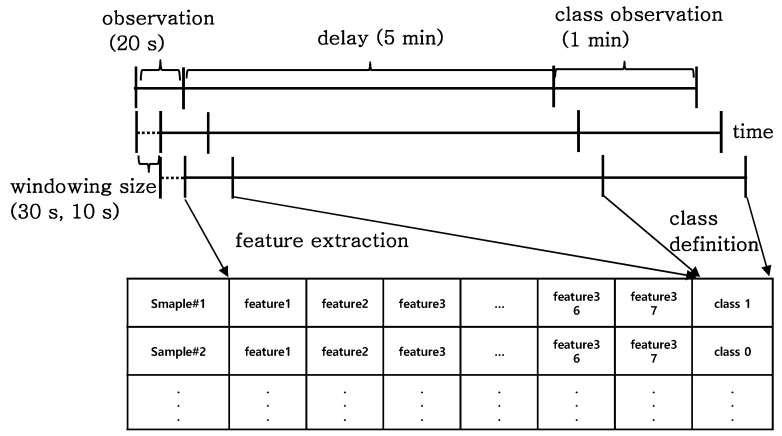
Sample generation process with an observation, delay, and class observation period. The observation period is how long feature generation can be observed, the delay is how far future events can be predicted, and the class observation is how long the event can be recognized.

For real-time forecasting, data samples were continuously generated through windowing, as shown in Figure 1. We attempted two choices for the length of windowing interval, 30 and 10 s, and compared their respective prediction results. As the windowing interval decreased, more samples were generated, which helped to examine the data in a fine grain.

**Figure 2:**
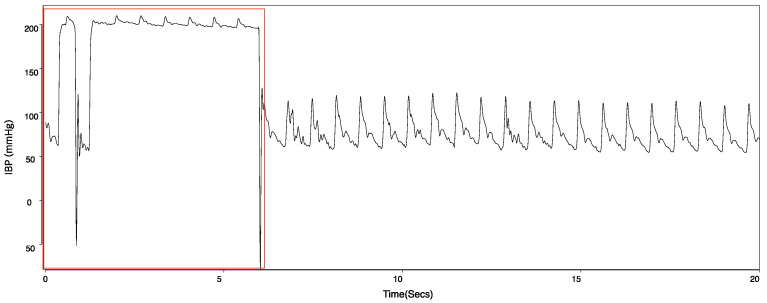
Artifacts marked in red on the time series of IBP.

Vital signs, as a form of time series through continuous monitoring, may display artifacts and noises due to electronic device errors, intraoperative events, or external pressure, as shown in Figure 2. To exclude artifacts and noise, we developed a criteria and excluded the samples that can satisfy various conditions. For example, the feature observation period and the class observation period, of which the maximum value exceeds 200 and the minimum value is under 20, were excluded. The case where the difference between the maximum and minimum during the feature observation or class observation is <30 conformed to an artifact. The difference between continuous values of 30 or less also conformed to artifacts. These slight variations for IBP occurred when the external pressure was applied to patients, usually to measure non-invasive blood pressure (NIBP) with cuffs. 

## 4. Methodology

### 4.1. Feature Engineering

We proposed a systematic feature engineering process using domain knowledge. The proposed feature engineering process is not specific to only one vital sign, but can generally be applied to any vital sign signal. 

The feature can be extracted in terms of the time domain and the frequency domain. The extensive feature engineering on the data observation period provides a hint for future events. To extract abnormality in values and their distribution, descriptive statistical analysis and peak analysis were both applied accordingly. The abrupt changes through change analysis were also captured. 

### 4.2. Descriptive Analysis

Through descriptive statistical analysis, the representative values were selected through the mean, minimum, and maximum. The dispersion metrics describe the size of the distribution of values. The dispersion metrics include the range, variance, standard deviation, and inter-quartile range (IQR). To explain the shape and symmetry of data distribution, skewness and kurtosis were used as representative metrics. Skewness is a statistic which can indicate the degree of asymmetry of a distribution. If the distribution is symmetrical, such as a normal distribution or a T distribution, the skewness is 0. The skewness of a distribution with a long tail to the right and that to the left denote positivity and negativity, respectively. The kurtosis describes the weight of the tails of data distribution compared to standard normal distribution. The root sum square (RSS) was adopted by taking the square root of the sum of the squares of all the data points. RMS takes the square root of the arithmetic mean square of data points. These metrics represent the data as representative values. RSS implies the signal strength, while the RMS indicates the average of RSS.

### 4.3. Peak Analysis

**Figure 3:**
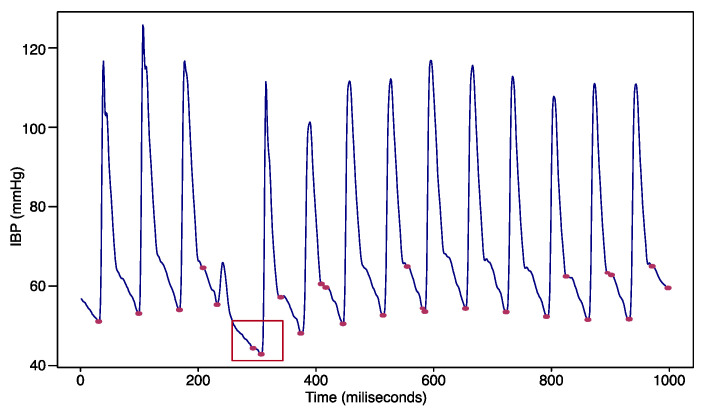
Downward peaks detected with a certain threshold.

The peak analysis aims to find the location of the local maxima or the minimum of a signal, and sorts the peaks by height, width, or prominence. Since our goal was to detect hypotension event, we defined the peak as the downward-sloping portion below 65, as marked in red in Figure 3. The statistical features on the peak detection results can be derived by the number of peaks, the mean, the standard deviation for the peak interval, the mean, the maximum, the minimum, the standard deviation for the peak value, and the crest factor. The crest factor shows the ratio of peak values to other values and represents the degree to which the peak is abnormal.

Figure 3 demonstrates that the peaks can characterize the cyclic patterns, even though the patterns seem apparently similar to each other. The bounding box area shows different patterns with a low peak and a downward peak as well. Two peak points in the downward peak appear consecutively, as marked in red in Figure 3. 

As demonstrated in Figure 3, peaks are useful to characterize cyclic patterns, even when they appear similar to one another. The bounding box area in red in Figure 3 shows different patterns from other time points. Two peak points in the downward peak appeared consecutively compared to other peak points.

### 4.4. Change Analysis

**Figure 4:**
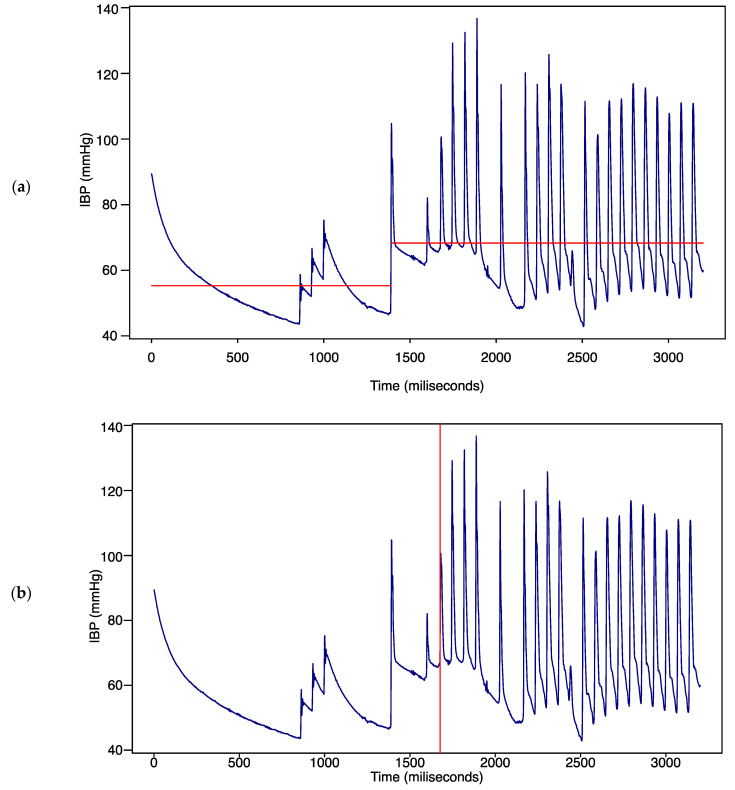
Change points analysis results. (**a**) Mean change; (**b**) variance change.

In the change analysis, the changes in mean and variance were detected. The change detection algorithm partitions a signal into adjacent segments where a statistic, such as the mean and the variance, is constant within a segment. To be more specific, the algorithm partitions the data into two parts and calculates the sum of the residual error of each part from its local mean. After detecting change points, the statistics, such as the number of changes in the mean, variance, and mean variance of blood pressure values, were accordingly derived. The red line in Figure 4 depicts the time point at which the mean changes (Figure 4a) and the time points at which the variance changes (Figure 4b). 

### 4.5. Frequency Analysis

**Figure 5:**
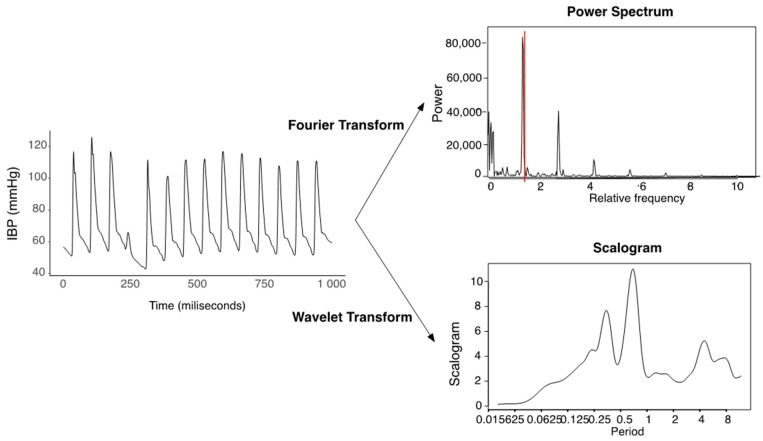
Fourier transform and wavelet transformation of the time domain signal.

The waveform data recorded in the time domain can be transformed into the frequency domain, as shown in Figure 5. The frequency analysis extracts major frequencies in forming the time series. The frequency analysis was divided into Fourier transform and wavelet transform. The spectrum through the Fourier transform, displaying the power, indicates how much a given frequency contributes to the signal. We used the fundamental frequency with the highest power and other frequencies which follow the fundamental frequency. The frequencies with the top three powers were used as features. 

In the wavelet transform, a wavelet, i.e., an oscillation form, was convolved with time-series data by scaling the wavelet and shifting into timelines. 

Wavelet families include various mother wavelets that can be applied differently depending on domains. The Morlet parent function can identify oscillated patterns.

Wavelet transform is a form of time–frequency representation. It gives the coefficients of scaling and shifting coefficients. The baseline of the signal’s scalogram is extracted through continuous wavelet transform. The scalogram value represents how much a wavelet scaled by a scale contributes to a signal at a certain time. We derived 10 scale values with the top scalogram values as features. The transformation of the time domain data into the frequency domain is shown in Figure 5. At the right upper panel in Figure 5, the periodogram from FFT shows the fundamental frequencies that lay at 0.02 and its multiples in terms of the relative frequency. 

The scalogram at the right bottom panel indicates the absolute value of the continuous wavelet transform of an IBP time series, plotted as a function of scale and power. Wavelet algorithm changes the wavelet scale and checks how much the scaled wavelet fits to the signal. It gives the contribution of each scale to the total energy of the signal. 

The 36 aforementioned features are listed in Table 2 below.

### 4.6. Model

**Figure 6:**
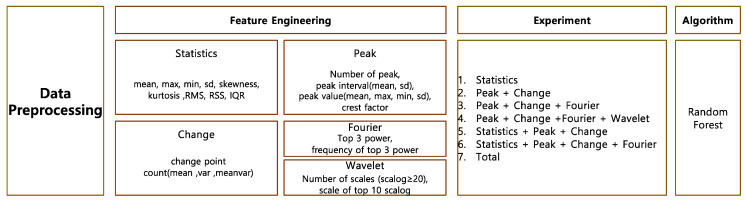
Research framework to build the hypotension prediction model.

We then applied machine learning to extract the features. We adopted the sophisticated model on account of numerous features. Random forest is a machine learning technique proposed by [22] and is one of the ensemble learning methods used for classification and regression analysis. In a random forest model, several decision trees are constructed, and each tree individually learns the sampled data using bagging with different sets of features. Bagging is a method used to sample datasets by allowing duplicates. Then, the results of classification are voted on, and the result that receives the most is determined as the final classification result. This is effective for large data processing and has the advantage of improving model accuracy by avoiding the overfitting problem. A random forest was constructed for each extracted feature combination. The number of decision trees of random forest was designated as 100. Figure 6 presents the overall framework of our model.

## 5. Experiment and Results

### 5.1. Data Collection

Our dataset collected vital signs, EMR, and anesthesia record from adult patients (age ≥18 years) who underwent laparoscopic cholecystectomy under general anesthesia at Soonchunhyang University Bucheon Hospital, Bucheon City, Republic of Korea between 30 December 2019 and 31 October 2000. The vital signs were collected using the vital recorder [21]. Data collection was approved by the Soonchunhyang Bucheon hospital review board (approval No. SCHDB_IRB_2011-11-015). Informed consent was obtained from all subjects or their legal guardians. All methods were performed in accordance with the relevant guidelines and regulations.

### 5.2. Experiment Results

The hypotension prediction model was built under a different feature set, as shown in Figure 6. Our dataset had an imbalance problem with far less hypotensive samples than normal samples. To resolve the class imbalance problem, the most widely used methods are up-sampling and down-sampling. Up-sampling upsizes the small class at random, while down-sampling downsizes the large class at random.

**Figure 7:**
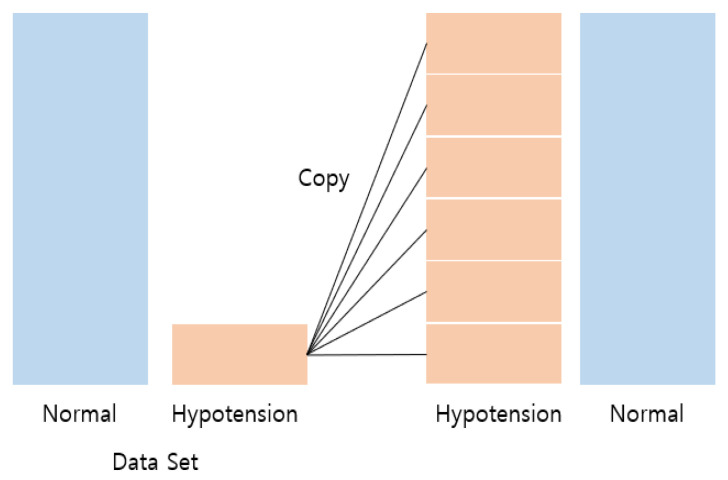
Up-sampling of hypotension class to resolve the class imbalance.

To overcome this imbalance, the data for the minor class were augmented by up-sampling the training dataset. Up-sampling copies the data from the low-quantity class as much as the data from the high-quantity class to make the distribution of the classes the same. Up-sampling was performed by merely copying the hypotension samples for as many normal samples, as shown in Figure 7. Up-sampling processing was only performed in training data, but the validation dataset was kept as original. 

**Figure 8:**
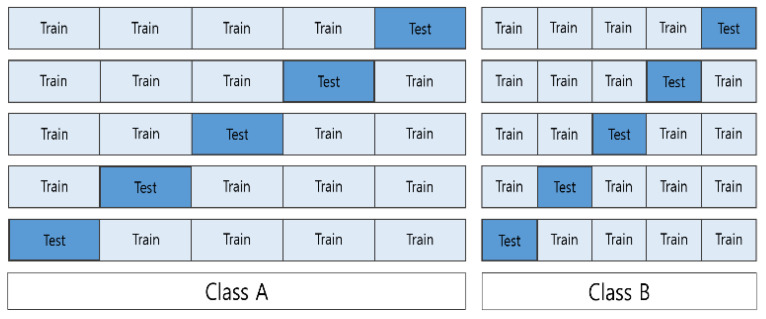
Visual representation of cross validation.

Stratified k-fold cross validation was performed to evaluate the model. In k-fold cross validation, the data were divided into k splits, k-1 splits were used as the train set, and the remaining one split was used as the test set. k-fold is used when the data are independent and have the same distribution. For the data in this study, stratified k-fold was used instead of k-fold, because the distribution of each class was not the same. Stratified k-fold cross validation performs k-fold while maintaining the distribution of classes, as shown in Figure 8. In this study, we set k to 5.

Accuracy for all classes and precision and recall for the hypotension class were used as the model performance indicators. Each expression is as follows. The hypotension is the same metric of sensitivity.

(1) $$Accuracy=\frac{TP+TN}{TP+FN+FP+TN}$$

(2) $$Precision=\frac{TP}{TP+FP}$$

(3) $$Recall\;\left(Senstivity\right)=\frac{TP}{TP+FN}$$

*TP*, *TN*, *FP*, and *FN* represent the true positives, true negatives, false positives, and false negatives, respectively. As indicated by the performance, precision and recall of hypotension class were primarily used. Due to the sample imbalance, the metric should focus on the minor class. The precision and recall of hypotension class were presented first and the accuracy was presented as an overall metric. 

The performances according to the feature sets are listed in Table 3. The results in Table 3 show that the fundamental frequencies and the Morlet wavelet, which captures the oscillation patterns, are both effective in improving characterization between the hypotension and normal class. The accuracy was as high as 0.974, but precision and recall for the positive class (hypotension) were rather low. This shows that the model works better with the normal class than with the hypotension class. The model was trained to precisely detect the hypotension and, as a result, it misses a significant portion of the hypotension, consequently yielding a low recall. 

To improve the performance, we modified the machine learning algorithm by adding different class weights to the cost function of the algorithm. Various methods were used to assign the weight onto the class as shown in Table 4. The balanced method involves adding weight in reverse order to the class distribution. The balanced subsample calculates weights which are inversely proportional to the class frequency based on bootstrap samples. We could improve the recall when adjusting the weight assigned to each class, but should compromise the precision metric. Thus, we kept the original normal without the weight assignment.

**Figure 9:**
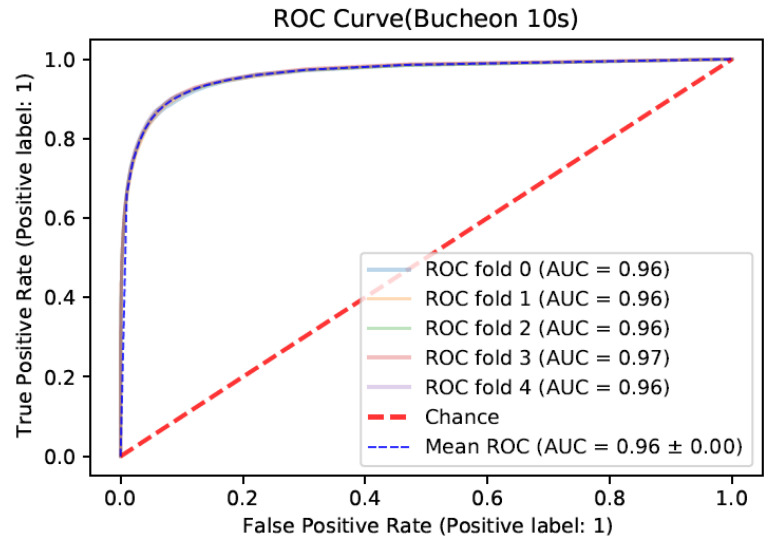
Receiver operating characteristic curves for each fold.

We also checked the receiver operating characteristic (ROC) curves for the best performed model in Figure 9. The ROC curves are consistently close to the ideal point which is (0, 1) for all cross-validation sets. As shown in Figure 9, the specificity relating to the normal class, calculated as the 1-*x* axis value (False Positive Rate), is very close to 1. This is because most of the samples are normal and the algorithm works well for this major class. 

**Figure 10:**
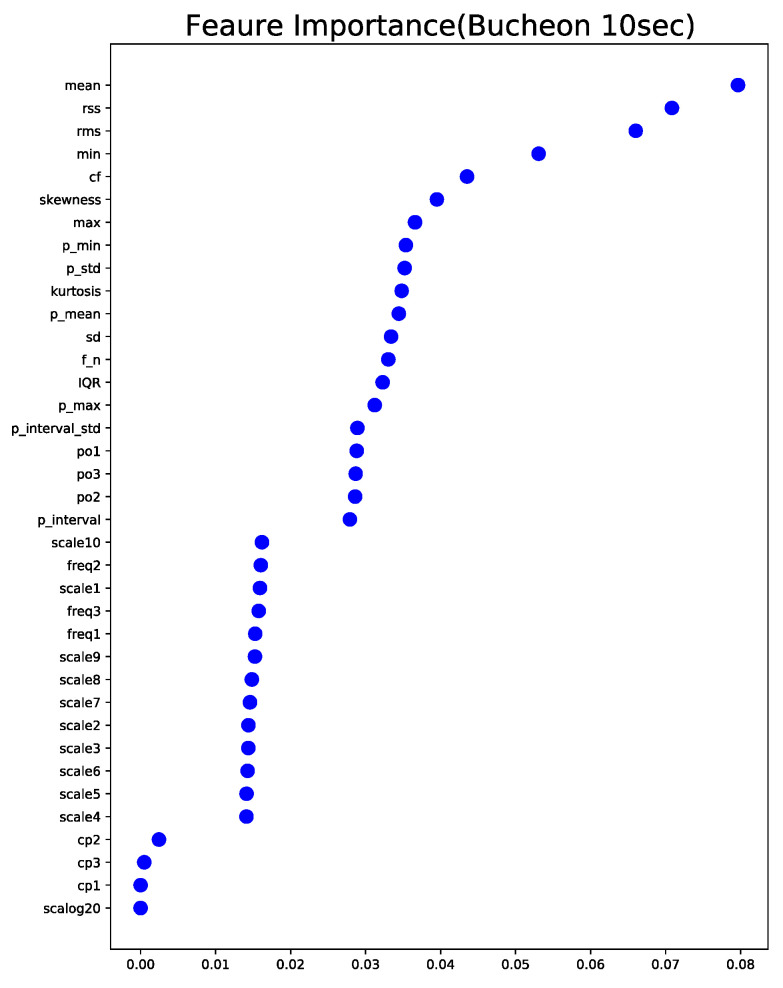
Feature importance plot.

To build an explainable machine learning algorithm, we assessed the impact of any given variable on the performance using feature importance. Feature importance is computed based on how important any given feature is to aid in the classification process when the classifier is built, determined by its effect on the performance measures. Gini importance is computed from the random forest structure. As shown in Figure 10, the most important features are listed as mean, RSS, RMS, and min of IBP. In terms of feature groups, the statistical feature set, the peak analysis feature set, the frequency analysis feature set, and the change analysis feature set were found to be important in that order.

### 5.3. Exploratory Analysis

Table 5 lists the vital signs according to hypotension and non-hypotension. All vital signs, except for the number of changes in the mean, were found to be significantly different. Overall, the IBP of the hypotension class is lower than that of the non-hypotension class. However, its skewness is higher. The IBPs of hypotension patients reach higher peaks than the non-hypotension class, and the peak values of the hypotension class are lower than those of the non-hypotension class. In addition, the peak values of the hypotension class have rather larger deviation than the normal class. The frequency of the hypotension class is higher than the non-hypotension class. This implies that IBP right before hypotension exhibits high vibration. The wavelet’s scales of the hypotension class are lower than those of the non-hypotension class. This implies that more sharp oscillations occur in the hypotension class compared to the non-hypotension class.

### 5.4. Comparison Analysis with Another Dataset

We performed an extra experiment with another dataset to verify the universality of our model. The public data from Seoul National University Hospital include all 6388 cases published in VitalDB whereby arterial pressure waveform monitoring was performed under general anesthesia. Those who are under the age of 18, weigh less than 30 kg or more than 140 kg, or who are less than 135 cm or more than 200 cm in height were excluded. In addition, the data cover 3278 files, excluding cases of transplant surgery, heart surgery, and vascular surgery. Like the data from Bucheon Hospital, it is recorded in units of 100 Hz. In this paper, only 983 were used for comparison, i.e., 30% of the data from Seoul National University Hospital. We found that the performance of our model for this dataset decreased, especially in terms of precision, as shown in Table 6. 

The data from Seoul National University Hospital performed worse than the data from Soonchunhyang University Bucheon Hospital. This appears to be due to the difference in the type of surgery between the two datasets. In the case of the Seoul National University Hospital data, all surgeries, except for transplant surgery, heart surgery, and vascular surgery, were included, whereas the data from Soonchunhyang University Bucheon Hospital were only for laparoscopic cholecystectomy. In addition, although the Seoul National University Hospital data had a larger number of samples than the Bucheon Hospital data, the event imbalance was more severe. Based on 30 s, the ratio of hypotension samples over entire samples for Bucheon hospital was 4.7% (11,956/240,314) and the ratio for Seoul hospital was 1.3% (3559/260,683).

## 6. Discussion and Conclusions

Currently, several studies that predict the amount of stroke, heart failure, and hypotension using vital signs during surgery have been published [15,16,18,19]. In the near future, results of these studies may be adopted as useful diagnostic tools, enabling an immediate reaction to hemodynamic changes and improving perioperative prognosis. 

The present authors conducted a study to predict the occurrence of hypotension 5 min in advance using vital signs. For that, we proposed a systematic feature engineering to build a real-time prediction model for hypotension in the operation room. This forecasting problem is quite challenging compared to diagnosis that detects high-risk patients at current. In particular, the forecasting problem that specifies the event occurrent time is very difficult to advance the predictable time. In this work, we challenged this problem through a systematic feature engineering and machine learning algorithm.

To process this problem, we tried to set up more a realistic condition than previous works. We included any hypotension, while previous works included the hypotension events that last for long time. One-off occurrences are more difficult to detect because there may be less precursor symptoms. In addition, we doubted whether previous works focus on the first point rather than following points during the hypotension. Any samples that embed hypotension during the observation and the delay should be deleted because they may give hints. 

For more information, we performed the comparison between the patients who suffer hypotension or not. Appendix A Table A1 lists the clinical characteristics of patients, including electronic medical record and laboratory data. The only age among demographics and anesthesia time, operation time, crystal fluid amount, blood loss, and anesthesia method among operation-related variables recorded in EMR differed significantly between hypotension and normal groups. Among the preoperative test results, most variables such as Hb, Hct, Plt, PT, INR, aPTT, AST, ALT, Alb, Na, K, and Cl have significantly lower values of hypotensive patients than those of normal patients. Glc, BUN, and Cr did not differ significantly and had no clinical implication. Among preoperative laboratory test results, chloride concentration differed significantly between the groups. Among past disease records, valvular heart disease, Diabete smellitus, HbA1c, and cerebrovascular disease showed a significant difference between normal and hypotension groups. The presence of this disease is found to significantly increase the risk of hypotension.

From the current experiment, we could identify several future research directions. 

Our problem is highly imbalanced for the hypotension class; thus, the model tends to be fitted to the normal class. As a consequence, it is hard to achieve good performance for the hypotension class. More specifically, our model does not cover hypotension samples, resulting in low recall. The low recall indicates that many patients who suffer hypotension later show no difference 5 min later compared to normal patients. This arguably suggests that the 5 min delay was too long, or that our feature engineering was insufficient. In future work, we will compromise the delay by checking the time point when differences between hypotension class and normal class are maximized. 

From the feature importance, we found that the IBP values themselves were lower in hypotension than in the normal class. From this observation, more sophisticated statistical features can improve the performance. *p*-Values corresponding to a certain one-side test statistic will tell the difference in the distributions of IBP in normal and hypotension classes. These *p*-values indicate how a large portion of the data is lower than the threshold. We aim to vary the threshold to improve the performance. 

Data were generated with windows of 30 s and 10 s, and features were extracted accordingly. The shorter the windowing interval, the better the performance. Furthermore, the model using all the features among the feature combinations showed the best performance. For future work, we will generate samples with the windowing interval in small units, such as 1 s. Furthermore, we will vary the observation and class observation period and check the performance. The best combination will be derived through the experiment. 

Lastly, we will also apply other algorithms, such as deep learning on raw data, or other assemble methods, such as XGboost or stacking based on the same feature sets.

## Figures and Tables

**Table 1:** The number of normal samples and hypotension samples with different windowing interval.

Interval	Normal Samples	Hypotension Samples
30 s	240,314	11,956
10 s	721,020	35,887

**Table 2:** Details of the feature set.

Category	Features	Number of Features
Statistics	Mean of blood pressure	9
	Max of blood pressure	
	Min of blood pressure	
	Sd of blood pressure	
	Skewness of blood pressure	
	Kurtosis of blood pressure	
	RMS of blood pressure	
	RSS of blood pressure	
	IQR of blood pressure	
Peak	Number of peak	11
	Mean of peak interval	
	Sd of peak interval	
	Mean of peak value	
	Max of peak value	
	Min of peak value	
	Sd of peak value	
	Crest factor	
	The number of changes in mean	
	The number of changes in var	
	The number of changes in mean-var	
Fourier	Top 3 power	6
	Frequency of top 3 power	
Wavelet	Top 10 scales with high scalog values	10
Total		36

**Table 3:** Prediction results according to different windowing intervals and different feature sets.

Feature Set	Windowing					
	30 s			10 s		
	Accuracy	Precision	Recall	Accuracy	Precision	Recall
Statistics	0.961	0.679	0.316	0.967	0.792	0.403
Peak + Change	0.958	0.646	0.269	0.963	0.750	0.338
Peak + Change + Fourier	0.963	0.759	0.335	0.971	0.873	0.451
Peak + Change + Fourier + Wavelet	0.964	0.780	0.345	0.972	0.891	0.468
Statistics + Peak + Change	0.963	0.745	0.339	0.970	0.861	0.444
Statistics + Peak + Change + Fourier	0.965	0.775	0.372	0.973	0.887	0.491
Total	0.966	0.970	0.379	0.974	0.904	0.511

**Table 4:** Prediction results according to different weights on the classes.

Class Weight	Windowing					
	30 s			10 s		
	Accuracy	Precision	Recall	Accuracy	Precision	Recall
balanced	0.964	0.795	0.326	0.972	0.914	0.461
balanced subsample	0.964	0.800	0.326	0.972	0.914	0.462
6:4	0.966	0.789	0.388	0.974	0.901	0.515
7:3	0.965	0.764	0.393	0.974	0.893	0.519
8:2	0.965	0.757	0.400	0.974	0.884	0.523
9:1	0.965	0.739	0.408	0.974	0.867	0.526

**Table 5:** Clinical patient characteristics in terms of vital signs.

Features	Non-Hypotension		Hypotension		*p*-Value
	Mean	Standard Deviation	Mean	Standard Deviation	
mean	84.403	12.810	65.480	10.956	<0.001
min	60.737	10.545	44.821	9.254	<0.001
max	124.119	18.869	105.325	17.668	<0.001
sd	17.528	4.873	16.007	4.485	<0.001
skewness	0.717	0.250	0.917	0.319	<0.001
rms	86.299	13.085	67.514	11.215	<0.001
rss	172,598.647	26,169.658	135,027.662	22,429.642	<0.001
IQR	28.128	10.679	22.535	8.861	<0.001
kurtosis	−0.678	0.841	−0.252	1.130	<0.001
p_n: number of peaks	30.221	23.457	52.457	30.431	<0.001
p_interval:means of peaks interval	81.605	67.791	45.741	30.487	<0.001
p_interval_std: standard deviation of peak intervals	47.246	79.895	25.111	29.733	<0.001
p_mean: mean of peaks	58.673	4.928	49.040	10.455	<0.001
p_max: maximum of peaks	62.625	3.514	56.908	11.508	<0.001
p_min: minimum of peaks	55.299	6.916	42.889	10.630	<0.001
p_std: standard deviation of peaks	2.052	1.766	3.927	2.327	<0.001
Cf: crest factor	1.442	0.111	1.566	0.154	<0.001
cp1: number of mean changes	1.000	0.002	1.000	0	0.699
cp2: number of variance changes	0.257	0.437	0.282	0.450	<0.001
cp3: number of mean and variance changes	0.991	0.095	0.987	0.112	<0.001
freq1: first strongest frequency	1.270	0.350	1.321	0.471	<0.001
freq2: second strongest frequency	1.271	0.349	1.323	0.469	<0.001
freq3: third strongest frequency	1.273	0.350	1.327	0.467	<0.001
po1: power of freq1	44,614.235	29,670.556	34,609.313	24,780.054	<0.001
po2: power of freq2	44,543.150	29,603.424	34,514.360	24,627.371	<0.001
po3: power of freq3	44,427.961	29,481.031	34,375.202	24,425.994	<0.001
scale1: the first largest scales of wavelet	0.785	0.288	0.775	0.426	<0.001
scale2: the second largest scales of wavelet	0.787	0.290	0.777	0.431	<0.001

**Table 6:** Verification results of the proposed model for other dataset.

Windowing					
30 s			10 s		
Accuracy	Precision	Recall	Accuracy	Precision	Recall
0.989	0.652	0.441	0.992	0.764	0.540

## Data Availability

Sample data is available on http://aibig.sch.ac.kr/data/listPageDATAHealthcare.do (accessed on 10 April 2022).

## Appendix A

**Table A1:** Clinical patient characteristics in terms of demographics, operation records, preoperative test results, and past disease history (% for discrete variable or standard deviation for continuous variable).

Characteristics		No Hypotension (*n* = 347)	Hypotension (*n* = 541)	*p*
Demographics				
Sex (Male)		182 (52.4%)	304 (56.2%)	0.306
Age		58.75 (15.04)	64.23 (15.15)	<0.001 *
Wt		62.34 (16.71)	61.53 (18.32)	0.507
Ht		154.31 (36.22)	152.71 (39.43)	0.541
BMI		23.00 (6.81)	22.59 (7.46)	0.404
ANE.Time		246.48 (133.48)	270.44 (174.82)	0.030 *
Operation Time		177.87 (119.72)	203.90 (178.96)	0.017 *
Crystal (mL)		894.81 (742.36)	1184.80 (1187.60)	<0.001 *
Colloid(mL)		239.39 (287.54)	274.62 (295.75)	0.080
Blood loss		201.06 (296.78)	373.76 (732.39)	<0.001 *
Urine. output		473.90 (968.22)	403.19 (548.59)	0.166
ASA	1	94 (27.1%)	88 (16.3%)	<0.001 *
	2	129 (37.2%)	192 (35.5%)	
	3	112 (32.3%)	201 (37.2%)	
	4	12 (3.5%)	54 (10.0%)	
	5	0 (0.0%)	3 (0.6%)	
	6	0 (0.0%)	3 (0.6%)	
EM		71 (20.5%)	175 (32.3%)	<0.001 *
Anesthesia Method	BPB	0 (0.0%)	1 (0.2%)	<0.001 *
	BPB_Volatile	0 (0.0%)	1 (0.2%)	
	CSE	0 (0.0%)	1 (0.2%)	
	MAC	0 (0.0%)	2 (0.4%)	
	spinal	5 (1.4%)	8 (1.5%)	
	TIVA	195 (56.2%)	171 (31.6%)	
	volatile	147 (42.4%)	357 (66.0%)	
Preoperative Test				
Hb		10.03 (5.52)	8.22 (5.92)	<0.001 *
Hct		29.83 (16.32)	24.50 (17.63)	<0.001 *
Plt		197.13 (134.62)	164.36 (140.36)	0.001 *
PT		10.25 (5.40)	8.99 (6.39)	0.002 *
INR		0.79 (0.42)	0.69 (0.50)	0.004 *
aPTT		27.18 (14.72)	23.91 (17.26)	0.004 *
AST		21.12 (17.61)	18.01 (17.89)	0.011 *
ALT		19.10 (19.65)	14.93 (17.92)	0.001 *
Alb		3.00 (1.80)	2.47 (1.92)	<0.001 *
Glc		71.67 (62.64)	64.42 (71.44)	0.122
BUN		13.40 (10.95)	12.19 (12.15)	0.134
Cr		1.97 (9.75)	1.43 (7.69)	0.357
Na		110.41 (56.63)	93.87 (65.06)	<0.001 *
K		3.27 (1.73)	2.80 (1.99)	<0.001 *
Cl		82.88 (42.61)	70.80 (49.13)	<0.001 *
History of Diseases				
HBsAg		17 (6.2%)	10 (2.7%)	0.050 *
RPR		4 (1.5%)	2 (0.5%)	0.441
Hypertension		150 (43.2%)	271 (50.1%)	0.054
Atrialfibrillation		16 (4.6%)	29 (5.4%)	0.734
Coronary artery disease		18 (5.2%)	38 (7.0%)	0.338
Angina pectoris		11 (3.2%)	18 (3.3%)	1.000
Myocardial infarction		3 (0.9%)	8 (1.5%)	0.620
Congestive heart failure		5 (1.4%)	16 (3.0%)	0.221
Valvular heart disease		3 (0.9%)	16 (3.0%)	0.062 *
Asthma		15 (4.3%)	36 (6.7%)	0.190
COPD		8 (2.3%)	20 (3.7%)	0.337
Interstitial lung disease		1 (0.3%)	1 (0.2%)	1.000
Hepatitis		8 (2.3%)	6 (1.1%)	0.263
Liver cirrhosis		9 (2.6%)	12 (2.2%)	0.894
Viral carrier		4 (1.2%)	3 (0.6%)	0.552
Fatty liver		1 (0.3%)	0 (0.0%)	0.823
HBV		12 (3.5%)	8 (1.5%)	0.088
HCV		4 (1.2%)	4 (0.7%)	0.786
Alcoholic		4 (1.2%)	5 (0.9%)	1.000
Autoimmune		0 (0.0%)	1 (0.2%)	1.000
Acute kidney injury		4 (1.2%)	7 (1.3%)	1.000
Chronic kidney injury		19 (5.6%)	26 (4.8%)	0.237
End stage renal disease		16 (4.6%)	30 (5.5%)	0.647
Diabetes mellitus		77 (22.2%)	178 (32.9%)	0.001 *
HbA1c		1.42 (2.80)	2.04 (3.20)	0.003 *
Thyroid disease		17 (4.9%)	18 (3.5%)	0.452
Myasthenia gravis		0 (0.0%)	1 (0.2%)	1.000
Morbid obesity		2 (0.6%)	2 (0.4%)	1.000
Epilepsy		2 (0.6%)	2 (0.4%)	1.000
Cerebrovascular disease		14 (4.0%)	42 (7.8%)	0.037 *
Cerebral aneurysm		8 (2.3%)	11 (2.0%)	0.971
Dementia		6 (1.7%)	15 (2.8%)	0.440

Note: * *p* < 0.05.
